# Four Hunterian Lectures and Bristol–1990

**Published:** 1991-06

**Authors:** N. M. P. Clarke, R. Miller, S. Nicholson, D. J. A. Scott, J. R. Farndon

**Affiliations:** Consultant Orthopaedic Surgeon, Southampton General Hospital; Senior Registrar South West Region; Lecturer in Surgery University of Bristol; Senior Registrar South West Region; Professor of Surgery University of Bristol


### Four Hunterian Lectures and Bristol — 1990

**N.M.P. CLARKE ChM, FRCS**
Consultant Orthopaedic Surgeon, Southampton General Hospital
**R. Miller MD, FRCS**
Senior Registrar South West Region
**S. Nicholson MD, FRCS**
Lecturer in Surgery University of Bristol
**D.J.A. Scott MB, ChB, FRCS**
Senior Registrar South West Region
**J.R. Farndon BSc, MD, FRCS**
Professor of Surgery University of Bristol

In 1990 Bristol had the signal honour and distinction of being associated with four Hunterian Professors and their lectures. Mr Clarke was a graduate of Bristol University and carried out his research work in the University of Bristol. Mr Miller and Mr Scott carried out their research in Bristol and are currently Senior Registrars on the South West rotation. Mr Nicholson carried out his research work in the University of Newcastle upon Tyne before being appointed Lecturer in the Department of Surgery in the University of Bristol.

Mr Miller gave his Hunterian Lecture at a meeting of the Royal College of Surgeons of England on 23rd March 1990 in the College at Lincoln's Inn Fields.

Mr Nicholson's Lecture was given during a meeting of the British Association of Surgical Oncology at St Mary's Hospital, Paddington on 30th November 1990.

Mr Scott and Mr Clarke gave their Lectures at a meeting of the College which was held in the Bristol Royal Infirmary on 9th November 1990. The President and members of Council in numbers attended this meeting which was brought to a finale with a subscription dinner in the Great Hall of the University of Bristol.

### HUNTERIAN PROFESSORSHIPS

On 26th April 1799 the Court of Assistants of the Royal College of Surgeons of England met to consider a proposal from the Trustees of John Hunter's museum that the College should take over its custody.

The Court of the College made three resolutions:

1. "That ....... the museum of anatomical and morbid preparations of the late Mr John Hunter has been selected and made with extraordinary skill and judgement and that being carefully preserved it would become of great national benefit promoting and extending the knowledge of anatomy and surgery."
2. "That if Parliament think proper to purchase this valuable collection ......., this Court ....... will use their utmost endeavour to render it conducive to the advancement of anatomical and surgical science."
3. "That the Master and Wardens be requested to sign a copy of the above minutes and resolution and to communicate the same to the Trustees under Mr Hunter's will."

This resolution was delivered to Mr Everard Home, one of the Trustees, and in a letter from the Treasury Chambers dated 7th December 1799, Mr Charles Long confirmed that Parliament would purchase the collection and entrust its charge to the College. Their Lordships made several conditions which described upkeep and access to the collection and "*that a course of lectures not less than 12 in number upon comparative anatomy, illustrated by the preparations, shall be given twice a year by some member of the Surgeons' Company.*"

The museum remained at Castle Street for the time being but following a meeting of the Court on 23rd December 1799 they accepted responsibility of the museum and thanked the Lords of the Treasury for the honour of their confidence.

The Lectures were suspended between 1800 and 1809. The first Professors in 1810 were Everard Home in Comparative Anatomy and Sir William Blizard, Kt., in Surgery. Home's lectures in 1810 were:

1. On the general distribution of the preparations on comparative anatomy in the Hunterian Collection
2. On the structure of those parts of animals which are formed for the purpose of motion
3. On the inherent powers and various arrangements of muscular fibres
4. On the growth of skull and bone
5. Of the skeleton
6. Of joints
7. On the progressive motion of animals
8. Progressive motion on two legs
9. On the stomach
10. On the stomach
11. On the stomach
12. On the complex teeth.

Currently the Council of the College selects not less than 12 lectures and elects separate lecturers all of whom must be Fellows of the English College. All those to whom lectureships are awarded are styled Hunterian Professor during their year of office. A majority of these awards is given to the younger surgeons in training in recognition of a very high standard of research work advancing knowledge in surgical disciplines.

### A SCIENTIFIC APPROACH TO THE STAGED INVESTIGATION AND TREATMENT OF CONGENITAL DISPLACEMENT OF THE HIP

**N.M.P. Clarke ChM, FRCS**

### Experimental Studies

It has been observed that the capital femoral ossific nucleus in congenital dislocation of the hip (CDH) is abnormal in size and shape and is delayed in its appearance. A study of this epiphyseal dysplasia was performed in which it was postulated that abnormal loading in congenitally dislocated hips results in abnormal development of the epiphysis.

Evidence was sought to ascertain whether such an association could depend at least in part on a significant induced abnormality of the vascular tree in the dislocated femoral head which could thereby be responsible for the observed dysplasia of the bony epiphysis.

The following studies were performed:

1. The examination of the radiological topography of the capital femoral ossific nucleus in normal and congenitally dislocated hips in human infants.

2. An atraumatic hip dislocation in young hormonally sensitised rabbits was produced by splintage of one hind limb. The blood supply of the femoral head and developing bony epiphysis was then examined by microvascular injection techniques and histological sectioning.

The results of the radiological study showed that the observed hypoplasia and dysplasia of the capital femoral ossific nucleus is a constant finding in CDH.

The studies of the gross anatomy, topography of the blood supply and the bony epiphysis in normal and dislocated rabbits' hips revealed that:

1. There is an intimate association between epiphyseal ossification and vascular proliferation in normal and dislocated femoral heads.
2. Prior to the formation of the bony epiphysis within the cartilagenous femoral head the cartilage canal vasculature is end-arteriolar in nature. With the formation of the bony epiphysis an anastomosing vascular tree develops.
3. Deformity of the femoral head induced by dislocation resulted in eccentric ossification with a corresponding alteration in the pattern of vascular proliferation. This eccentric ossification was related to the altered area of contact (and thus joint stress transference) between femoral head and acetabulum which became postero-lateral in dislocated femoral heads.

The study showed that the blood supply at the arterial and arteriolar level remains normal in the atraumatically dislocated femoral head and that mechanical factors were not found to influence the normal development of the arteriolar blood supply during growth. However, the capillaries were found to proliferate within cartilage to produce ossification eccentrically. Additionally the cartilage canal vasculature of the femoral head prior to formation of the bony epiphysis is fragile and end-arteriolar in nature. With the appearance of the bony epiphysis an anastomosing vascular pattern develops which will be more resistant to compressive injury and resulting avascular necrosis.

### Ultrasound Studies

Ultrasound examination of the infant hip can reliably image the soft tissue structures and the cartilagenous femoral head. Dynamic examination with real time ultrasound allows hip instability to be depicted and replaces tactile interpretations. Sequential ultrasound studies can document the physiological resolution of neonatal hip instability that occurs in a proportion of cases and thus save over-treatment by splintage of many neonates. However, ultrasound can also identify those cases of hip instability that do not resolve and that do require treatment. An ultrasound examination of the hips at the age of two weeks will thus indicate the necessity for treatment.

Since hip location can subsequently be accurately documented while treatment is in progress management of hip instability by splintage can be monitored. Once hip location is obtained acetabular development may then be documented by the sonographic study of the cartilagenous acetabular labrum. Failure of early concentric femoral head reduction documented sonographically allows splintage to be abandoned before compressive vascular injury may occur.

The experimental studies have demonstrated the key alteration in vascular morphology that occurs with the development of the bony epiphysis.

The vascular proliferation that occurs prior to the commencement of ossification in the femoral head is seen with ultrasound before the bony epiphysis appears on the radiograph. It is postulated that surgical intervention in those dislocated hips that fail to reduce with conservative treatment is postponed until epiphyseal vascular proliferation occurs since the anastamosing circulation will sustain larger pressures and consequent injury. Therefore sonographic monitoring of established hip dislocation will allow delayed surgery to be timed in accordance with vascular maturation and the appearance of the bony epiphysis.

### A STUDY OF THE ROLE OF ANAL SENSATION IN THE CONTINENCE MECHANISM

**R. Miller MD, FRCS**

The aim of this study was to determine the possible contribution that anal sensation makes to the maintenance of anorectal continence and thereby improve the treatment of faecal continence.
The questions addressed were:

1. Is the anal canal in health sensitive to mechanical and thermal stimuli? Elsewhere in the body both are necessary for sensory discrimination between solid, liquid and gas.
2. Do patients with idiopathic faecal incontinence (IFI) have a sensory deficit to these stimuli in the anal canal?
3. Is there a demonstrable temperature difference between the rectum and anal canal as this is necessary for stimulation of thermal receptors?
4. Does spontaneous sphincteric relaxation allow equalisation of rectal and anal canal pressures permitting contact between rectal contents and anal mucosa? What is the minimum rectal distension that can induce this sampling response and is it abnormal in patients with IFI?
5. Is anal sensation modified by operations for anorectal incontinence?

### Methods

Mucosal electrostimulation (MES) was used to assess mechanosensitivity in the anal canal as at threshold levels this stimulus activates "touch" sensory receptors. A specially constructed water perfused thermode was used to determine thermal sensitivity. Thresholds to each stimulus were measured in the lower, middle and upper thirds of the anal canal as defined by water filled micro-balloon manometry.

To assess anorectal sampling a microtransducer catheter was used to measure pressures from the mid-anal canal and rectum. To study sampling in more physiological conditions a new technique was developed which allowed ambulatory anorectal manometric recording over prolonged periods of time. Lateral radiographs of the rectum filled with barium were used to measure anorectal angle and perineal descent.

### Results

1. In 45 control subjects the anal canal was found to be highly sensitive to MES (Median threshold 4–6 milliamps). In addition, the anal canal was very sensitive to temperature change with thresholds similar to those of the lip and face, the most sensitive areas of the body (threshold to warming stimulus – 0.5–0.8°C).
2. Thirty-two patients with IFI were studied. A severe sensory deficit was found in all three zones of the anal canal for both MES and temperature change. One third of the stimuli were not felt at all in the upper anal canal (MES 12 milliamps, thermal threshold 3.0- > 4.5°C).
3. The rectum was 0.1°C warmer than the upper anal canal, 0.2°C warmer than the middle and 0.4°C warmer than the lower third of the anal canal.
4. For the first time, anorectal sampling was seen to occur spontaneously in 16 of 20 normal controls studied. The median volume of rectal distension to induce the response was 10ml, much lower than previously thought. Sampling in 19 age and sex matched patients with IFI was found to be abnormal. Only four sampled spontaneously and a higher volume (40ml) was required to induce sampling in this group.
   In 15 ambulant controls, sampling was seen to occur spontaneously seven times per hour. In addition, the conscious awareness of the presence of flatus was associated with sampling in 80% of 144 recorded events.
5. Anal sensation in the upper anal canal as assessed by MES was found to improve following postanal repair (n = 13) and anterior sphincter plication (n = 7) with a return of thresholds to the normal range. Resting and "squeeze" anal canal pressures also improved but there was no change in anorectal angle.

### Conclusions

The anal canal in health is exquisitely sensitive. The sampling reflex allows this sensitive mucosa to come into frequent contact with rectal contents thereby constantly updating the CNS with sensory information regarding the presence and nature of rectal contents. This mechanism permits flatus to be passed at will whilst solid stool is retained.

Both anal sensation and sampling are defective in patients with IFI, accounting for their difficulties in discriminating solid stool from flatus and being unaware of defaecation.

The anal mucosa and internal anal sphincter should be preserved if at all possible when operating on the anal canal and efforts directed towards improving anal canal sensation in the management of idiopathic faecal incontinence.

### EPIDERMAL GROWTH FACTOR RECEPTOR MEASUREMENT IN PRIMARY HUMAN BREAST CANCER: A MARKER OF POOR PROGNOSIS AND LACK OF RESPONSE OF ENDOCRINE THERAPY

**S. Nicholson MD, FRCS**

Specific, high affinity, transmembrane receptors for epidermal growth factor (EGFr), a powerful mitogen for both normal and malignant mammary epithelial cells, have been demonstrated on primary human breast cancers. There were major differences in EGFr assay methodology between the various groups reporting this finding and there was no agreement on a clinically useful quantitative level of EGFr expression.

EGFr expression appeared to be inversely related to oestrogen receptor (ER) expression in human breast cancer raising the possibility that it may have prognostic implications.

The aims of the study were:

1. To compare methodology for EGFr analysis.
2. To develop a simple, inexpensive and reproducible screening assay for EGFr which was able to provide quantitative binding data and which was suitable for the analysis of small amounts of tumour tissue.
3. To correlate different methods of EGFr analysis, for example, radioligand assay and immunohistochemistry.
4. To examine mechanisms of EGFr over-expression by southern and northern analysis of tumour DNA and RNA respectively.
5. To study, in a large group of women with breast cancer, the association of EGFr expression with ER, Bloom & Richardson tumour grades, DNA ploidy, tumour size and axillary lymph node status; all well documented markers of tumour aggressiveness and prognosis.

### Methods

Over a five year period tumour biopsies were collected from 261 patients with operable breast cancer treated surgically and from 20 elderly (> 65 years) patients who underwent surgery after a failure of primary endocrine therapy.

Cell membrane specimens were prepared by tumour homogenization and differential centrifugation and analysed for EGFr using $I^{125}$ labelled EGF radioligand assays. Frozen tumour biopsies were also analysed for EGFr by an immunohistochemical method. ER was measured by dextran coated charcoal assay, with a cut-off value of 5fmol/mg for cytosolic ER.

Flow cytometric analysis of tumour material was also performed to determine DNA content. Southern and northern analysis of tumour DNA and RNA respectively were performed to examine cellular mechanisms of EGFr over expression in breast cancer.

Prospective patient follow-up over a six year period was performed to study the effect of EGFr expression of prognosis and response to therapy at relapse.

### Results

1. Multipoint radioligand assays for EGFr, analysed by the method of Scatchard, revealed two classes of binding sites in the majority of tumours expressing EGFr. Displacement assays underestimated the affinity of the high affinity site, thought to be biologically most relevant, compared with the saturation assays. The Kd (affinity constant) of the high affinity EGFr binding site derived from the latter was of the order 0.1 – 1n molar (median 0.63n molar).
2. Multipoint saturation assays revealed near complete occupation of available high affinity binding sites when the concentration of radiolabelled EGF approached 1n molar and when this single concentration was used in a screening assay the quantitative binding value for EGFr correlated with high effinity site capacity derived from multipoint saturation assays ($r = 0.99$, $p < 0.0001$). Statistical analysis of binding data allowed the determination of a cut-off value of 10fmol/mg for EGFr, which was used in subsequent analysis.
3. EGFr status determined by radioligand assay correlated with that determined by frozen section immunohistochemistry ($x^2 = 9.56$, $p < 0.0002$).
4. EGFr gene amplification determined by southern analysis was found to be a rare occurrence (~3% tumours) and therefore not a major reason for EGFr overexpression in human breast cancer. Tumours with high levels of EGFr did exhibit significant levels of EGFr messenger RNA on northern analysis.
5. EGFr were expressed on 95 of 261 primary tumours (36%). EGFr expression was inversely related to ER expression ($x^2 = 43.37$, $p < 0.0001$). Tumours expressing EGFr were more likely to be of high histological grade (poorly differentiated) ($x^2 = 10.8$, $p < 0.005$). EGFr expression was independent of tumour size, axillary lymph node status and DNA content.
   Patients with tumours expressing EGFr had a poor prognosis with shorter recurrence free ($x^2 = 13.96$, $p < 0.001$) and overall survival ($x^2 = 14.3$, $p < 0.001$, logrank). EGFr status was of particular prognostic value in patients in otherwise good prognosis subgroups where expression of EGFr led to a significant reduction in survival, eg:
   - axillary lymph node negative ($x^2 = 8.3$, $p < 0.005$, logrank) (p 0.01, Cox multivariate analysis)
   - Bloom and Richardson grade I and II tumours ($x^2 = 8.57$, $p < 0.005$, and $x^2 = 12.3$, $p < 0.001$ logrank, respectively)

   EGFr expression further stratified patients with ER negative tumours ($x^2 = 4.05$, $p < 0.05$).
   Patients with tumours expressing EGFr were more at risk of visceral relapse and less likely to respond to endocrine therapy compared with patients with tumours expressing ER ($x^2$ = for linear trends = 17.4, $p < 0.01$). Their disease progressed more rapidly on endocrine therapy compared with patients with EGFr negative tumours ($x^2 = 8.65$, $p < 0.005$, logrank).
   Tumours unresponsive to primary endocrine therapy in the elderly were significantly more likely to be EGFr positive than tumours from age matched controls treated surgically. EGFr expression in this group was associated with rapid tumour growth on endocrine therapy.

### Conclusions

One third of human breast cancers express EGFr. These are likely to be of high histological grade, ER negative and associated with a poor prognosis, particularly for patients with otherwise good prognostic markers. EGFr expression indicated likely visceral relapse and failure of endocrine therapy in those patients with recurrent breast cancer and in elderly patients treated by primary endocrine therapy. In patients with uninvolved axillary nodes EGFr expression might identify a subgroup of patients to receive adjuvant chemotherapy and in the elderly those who should proceed directly to surgical treatment. Such decisions can be made from results of an assay which is easily carried out and simple to establish.

### LOWER LIMB CRITICAL ISCHAEMIA – SELECTING PATIENTS FOR FEMORODISTAL BYPASS SURGERY

**D.J.A. Scott MB, ChB, FRCS**

Today most vascular surgeons base their selection for femorodistal (FD) bypass upon the combination of clinical examination, arteriographic and Doppler evidence of runoff. The use of non-reversed vein grafting and more distal bypass has increased the scope of possible limb salvage, but case selection has become more difficult. Peripheral resistance (PR) has recently gained acceptance as the best predictor of successful graft outcome.

The questions addressed were:

### (A) Pre-operative

1. Can a non-invasive peripheral resistance value be derived which will predict the operative peripheral resistance and subsequent outcome?
2. Is Intra-arterial digital subtraction arteriography (IA DSA) better at predicting PR than conventional arteriography?

### (B) Operative

3. Which haemodynamic parameters accurately predict successful femorodistal graft function?
4. Is the long saphenous vein (LSV) a potential cause of graft failure?

### (C) Post-operative

5. What are the best predictors of long term graft function?

### Method

A series of non-invasive tests were used: Doppler pressures/indices and Pulse Generated Runoff (PGR). A conventional arteriogram (CA) and IA DSA were performed in all cases. At the time of surgery PR and Graft Resistance (GR) were measured using the Bristol Doppler Flowmeter. Samples of the LSV were taken in excess of that required for bypass and distended to a pressure of 100mmHg for both light and electron microscopy. Post-operatively all grafts were followed up in the Vascular laboratory.

Forty-eight patients were studied prospectively and compared with a previous series of 40 patients for comparative results.

### Results

### (A) Pre-operative

1. Using multiple linear .regression (MLR) three resistance values were derived for grafts anastomosed to (i) a single calf vessel (R1), (ii) distal popliteal (R3) and (iii) irrespective of the level of the anastomosis (RO). The predicted PR values were then compared with the measured PR.

| | PR | R0 | R1 | R3 |
|---|---|---|---|---|
| **ARTERIOGRAPHY** | -0.43* | 0.45* | 0.55 | 0.55* |
| **PGR** | -0.60* | 0.59* | 0.96* | 0.56* |

Analysis Spearman Rank $r_S$ * $p<0.001$, \$ = NS

Using the appropriate equations in a subsequent prospective study of 13 grafts there was an excellent correlation of $r_s$ = 0.87, $p<0.001$.

2. Eighty-eight FD grafts were analysed (40 CA alone; 48 CA and IA DSA). Three widely used arteriogram scoring systems were correlated against the measured PR and subsequent graft outcome.

### (i) Distal Popliteal Grafts

There was a poor correlation between all three scores and the measured PR; in the IA DSA group there was a better correlation between the PR and subsequent graft outcome ($p<0.001$).

### (ii) Single calf vessel Grafts

All three scores failed to correlate with PR and graft outcome in both the conventional and IA DSA groups.

### (B) Operative

In a retrospective discriminant analysis of 48 (FD) grafts, the combination of a GR after papaverine of <1 PRU's and a retrograde flow (RF) of <33% had a sensitivity of 97% and a specificity of 83% for a successful outcome. These criteria were applied in a prospective study of 48 FD grafts.

### Results

3. The combination of a GR
4a) All the LSV showed varying degrees of intimal hyperplasia.
4b) Ten per cent of the veins had stenoses > 75% prior to implantation.
4c) Marked degrees of muscle fibrosis and hypertrophy were noted.
4d) The use of the Hall valvulotome caused extensive endothelial cell loss of > 75% in 55% of cases.

### (C) Post-operative

Eighty-one non-reversed femorodistal vein grafts have been followed up at regular intervals by Duplex scanning and Doppler pressures at rest and after exercise.

### Results

5a) The two year patency rates for grafts with arteriogram scores of <2 and >2 were 52% and 74% respectively (Lee-Desu $p<0.003$).
5b) Grafts with a PGR score of > 2 had one and two year patency rates of 79% and 64% respectively which were significantly higher than grafts with a PGR score of <2 of 14% ($p<0.002$).
5c) Grafts with resistances of <1, 1–2, >2 after the administration of papaverine had patency rates of 85%, 20% and 14% at one year ($p<0.001$).

### CONCLUSIONS

The introduction of IA DSA has improved the visualization of calf vessel runoff and the prediction of peripheral resistance. A non-invasive peripheral resistance value can now be derived using PGR which will accurately predict the measured peripheral resistance. These predictive factors plus operative measurements allow accurate prediction of graft function and therefore selection of patients for reconstruction or amputation. The long saphenous vein has been shown to be a potential source of graft failure and studies are now underway to predict the vein structure prior to surgery.

These abstracts of the work of the four Bristol Hunterian Professors in 1990 demonstrate the breadth, significance and value of research in surgical disciplines. It is hoped that elements of research will continue to be an integral part of surgical training and endeavour.

